# Chronic Corticosteroid Use Is Associated with Higher Perioperative Morbidity After Elective Primary Total Hip Arthroplasty

**DOI:** 10.3390/jcm15135057

**Published:** 2026-06-29

**Authors:** Assil Mahamid, Hamza Murad, Miri Elgabsi, Neev Tchernin, Aia Bowirrat, Feras Qawasmi, Dror Robinson, Mohammad Shehadeh, Mustafa Yassin, Muhammad Khatib

**Affiliations:** 1 Department of Orthopedics, Hasharon Hospital, Rabin Medical Center, Tel Aviv University, Tel Aviv 6997801, Israel; 2 Gray Faculty of Medical & Health Sciences, Tel Aviv University, Tel Aviv 6997801, Israel; 3 Division of Surgery, Hillel Yaffe Medical Center, Hadera 3820302, Israel

**Keywords:** total hip arthroplasty, corticosteroids, postoperative complications, bleeding, venous thromboembolism

## Abstract

**Background:** Chronic corticosteroids are commonly prescribed for autoimmune and inflammatory disorders, yet their impact on perioperative outcomes following elective total hip arthroplasty (THA) remains incompletely defined. This study evaluated the association between chronic corticosteroid use and postoperative complications and hospital outcomes after elective primary THA. **Methods:** We performed a retrospective cohort study using the National Inpatient Sample (2016–2021). Adult patients undergoing elective primary THA were identified using ICD-10-PCS codes. Chronic corticosteroid use was defined by ICD-10-CM code Z79.52. The primary outcome was any postoperative complication, including venous thromboembolism (VTE), major bleeding, acute kidney injury, myocardial infarction, stroke, or sepsis. Secondary outcomes included prolonged length of stay, high hospital charges, discharge to rehabilitation, and in-hospital mortality. Multivariable weighted logistic regression and 1:1 propensity score matching (PSM) was applied. **Results:** The weighted cohort represented approximately 600,000 hospitalizations, of which 0.91% involved chronic steroid use. Steroid users had a higher burden of comorbidities. After adjustment, chronic corticosteroid use was independently associated with increased odds of any postoperative complication (OR 1.32), major bleeding (OR 1.46), prolonged hospitalization (OR 1.26), discharge to rehabilitation (OR 1.06), and in-hospital mortality (OR 2.53). In the matched cohort (1079 pairs), steroid use remained significantly associated with overall complications (OR 1.84) and acute kidney injury (OR 2.10). **Conclusions:** Although uncommon, chronic corticosteroid use is associated with a clinically meaningful increase in perioperative morbidity after elective THA. These findings highlight chronic corticosteroid use as a marker of increased perioperative risk that warrants greater clinical recognition, and they provide hypothesis-generating evidence to inform future studies of perioperative management in this population.

## 1. Introduction

Chronic systemic corticosteroid therapy is a well-established risk factor for adverse outcomes following total hip arthroplasty (THA). Multiple large cohort studies have demonstrated significantly increased rates of periprosthetic joint infection (PJI), hospital readmission, and revision surgery among patients receiving long-term corticosteroids compared with non-users. Reported effect sizes for PJI range from 1.7 to 3.3, with the highest risks observed in individuals with underlying inflammatory conditions such as inflammatory bowel disease, particularly when steroid exposure occurs within the year preceding arthroplasty [1,2]. Chronic steroid users also exhibit higher 30- and 90-day readmission rates and an elevated likelihood of revision arthroplasty within the first postoperative year, reflecting both infectious complications and impaired bone and soft-tissue quality associated with prolonged corticosteroid exposure [3]. Beyond perioperative outcomes, long-term corticosteroid therapy has been linked to an increased lifetime risk of requiring hip arthroplasty for both fracture-related and non-fracture indications, with dose–response effects documented for non-fracture pathways [4].

Importantly, the exposure evaluated in the present study differs from short-term perioperative corticosteroid administration used for postoperative analgesia or antiemetic prophylaxis. Whereas previous studies have examined single-dose or short-course perioperative corticosteroids, the current analysis used ICD-10-CM code Z79.52 as a proxy for long-term current corticosteroid use and was limited to outcomes occurring during the index hospitalization. Randomized trials and meta-analyses consistently show that single-dose or short-course corticosteroids used for postoperative pain, inflammation, or nausea control do not increase rates of infection or other major complications in otherwise healthy arthroplasty patients [5,6]. Furthermore, although chronic corticosteroid users are susceptible to infectious complications, wound-healing disturbances, and readmission, most studies have not demonstrated a clinically meaningful increase in perioperative major bleeding or gastrointestinal hemorrhage attributable solely to chronic steroid exposure [7,8]. Several investigations have also reported modestly increased postoperative mortality among chronic steroid users undergoing THA, although interpretation is complicated by the substantial comorbidity burden in this population; meta-analytic estimates suggest approximately a 30% relative increase in 30-day mortality after adjustment for confounding factors [7,8,9].

Although previous studies have examined the relationship between chronic corticosteroid use and postoperative outcomes after total joint arthroplasty, contemporary nationally representative data focusing specifically on elective primary THA remain limited. Therefore, the purpose of this study was to evaluate the association between chronic systemic corticosteroid use and selected in-hospital outcomes following elective primary THA using the National Inpatient Sample. Specifically, we examined associations with postoperative complications, venous thromboembolism, infection, hospitalization metrics, and in-hospital mortality. By providing contemporary estimates from a large national cohort, this study aims to further characterize perioperative outcomes among patients receiving chronic corticosteroid therapy undergoing elective THA.

## 2. Materials and Methods

### 2.1. Data Source

This retrospective study utilized data from the National Inpatient Sample (NIS), the largest publicly available inpatient database in the United States. The NIS, developed by the Agency for Healthcare Research and Quality (AHRQ) as part of the Healthcare Cost and Utilization Project (HCUP), contains a stratified 20% sample of all hospital discharges nationwide, representing approximately seven million unweighted and over thirty million weighted annual hospitalizations. Hospital discharge weights (DISCWT) provided by HCUP were applied to generate nationally representative estimates. Data spanning 1 January 2016 through 31 December 2021 were included to capture the most recent six-year period available.

### 2.2. Study Population

Adult patients (≥18 years) undergoing elective primary total hip arthroplasty (THA) between 2016 and 2021 were identified using ICD-10-PCS procedure codes (0SR90J9, 0SR90JA, 0SR90JZ, 0SRB0J9, 0SRB0JA, and 0SRB0JZ). These codes have been widely used in prior administrative database studies evaluating primary THA and represent replacement of the right or left hip joint with synthetic substitutes. Chronic systemic corticosteroid use was identified using ICD-10-CM code Z79.52, which denotes long-term current use of systemic corticosteroids. This code was used as a proxy for chronic corticosteroid exposure; however, the NIS does not provide information regarding steroid dose, route of administration, duration, adherence, tapering schedules, indication for therapy, or whether corticosteroid treatment was continued or modified during the perioperative hospitalization.

### 2.3. Outcomes

The primary outcome was any postoperative complication, defined as the occurrence of one or more major adverse in-hospital events following THA: venous thromboembolism (deep vein thrombosis or pulmonary embolism), major bleeding (hemorrhage or transfusion), acute kidney injury, sepsis, myocardial infarction, or stroke. Secondary outcomes included each component complication analyzed individually, as well as prolonged length of stay (>75th percentile), high hospital charges (≥75th percentile), discharge to a rehabilitation facility, and in-hospital mortality. Complications were identified using ICD-10 diagnostic and procedural codes during the index hospitalization only; readmissions were not captured. Table 1 summarizes the ICD-10 codes used to define exposure, comorbidities, and postoperative complications.

### 2.4. Exposure Variables and Statistical Analysis

Patient-level variables included age, sex, primary payer, household income quartile, and comorbidities including hypertension, diabetes mellitus, dyslipidemia, obstructive sleep apnea, chronic anemia, alcohol abuse, chronic kidney disease, congestive heart failure, obesity, and chronic lung disease. Hospital-level characteristics included region, bed size, teaching status, and ownership type. Baseline demographic, clinical, and hospital characteristics were compared between patients with and without chronic corticosteroid use. Categorical variables were compared using Pearson’s χ^2^ tests, and continuous variables were summarized as weighted means ± standard deviations and compared using independent t-tests. Length of stay and total hospital charges were analyzed both as continuous variables and as dichotomized outcomes using the 75th percentile as the threshold for prolonged hospitalization and high hospital charges, respectively.

### 2.5. Missing Data

Missing data were minimal for most covariates (<0.2%). However, obesity and chronic lung disease demonstrated substantial missingness (>80%) and were therefore excluded from adjusted analyses but reported descriptively. Complete-case analysis was performed without imputation. Although exclusion of these variables may have resulted in residual confounding, particularly because chronic lung disease was more prevalent among chronic steroid users, the extent of missingness precluded reliable adjustment or imputation. Consequently, adjusted estimates should be interpreted in the context of potential residual confounding arising from incompletely captured comorbidities.

### 2.6. Statistical Analysis

Weighted multivariable logistic regression models were constructed to estimate adjusted odds ratios (ORs) and 95% confidence intervals (CIs) for postoperative outcomes associated with chronic corticosteroid use. Covariates included age, sex, primary payer, household income quartile, hypertension, diabetes mellitus, dyslipidemia, obstructive sleep apnea, chronic anemia, alcohol abuse, chronic kidney disease, congestive heart failure, hospital region, hospital bed size, teaching status, and ownership type. Obesity and chronic lung disease were excluded from adjusted analyses because of extensive missing data. For each outcome, the same prespecified set of covariates, selected a priori on the basis of clinical relevance and the prior literature rather than stepwise or other data-driven selection procedures, was entered simultaneously in a single-block logistic regression model; no outcome-specific variable selection was performed. Survey discharge weights (DISCWT) were applied to generate nationally representative estimates, consistent with HCUP-NIS recommendations.

Propensity score matching (PSM) was performed using 1:1 nearest-neighbor matching without replacement based on the logit of the propensity score. The propensity score model included the same demographic, clinical, and hospital-level covariates used in the multivariable regression analysis. Covariate balance was assessed using standardized mean differences (SMDs), with values below 0.10 considered indicative of adequate balance. A total of 1241 of 1265 chronic steroid users were successfully matched.

In accordance with HCUP-NIS recommendations, survey weights were applied only in regression analyses and not in the matched cohort. Statistical significance was defined as a two-sided *p*-value ≤ 0.05. All analyses were performed using Python (version 3.10).

## 3. Results

### 3.1. Patient Population

After applying inclusion and exclusion criteria, a total of 119,944 adult hospitalizations for elective primary total hip arthroplasty (THA) were identified in the 2016–2021 National Inpatient Sample (NIS). When weighted to national estimates, this corresponded to approximately 600,000 hospitalizations. Of these, 1091 (0.91%) involved patients with chronic corticosteroid use (ICD-10 code Z79.52).

### 3.2. Baseline Characteristics

Steroid users were slightly younger (63.8 ± 13.8 years vs. 65.1 ± 11.1 years; *p* = 0.0013) and more frequently female (60.8% vs. 54.8%; *p* < 0.001). They also had higher rates of hypertension (12.7% vs. 9.7%; *p* = 0.0011), obstructive sleep apnea (13.5% vs. 10.0%; *p* < 0.001), chronic anemia (8.4% vs. 5.4%; *p* < 0.001), chronic lung disease (14.1% vs. 7.3%; *p* < 0.001), and congestive heart failure (6.0% vs. 2.9%; *p* < 0.001). Minor but statistically significant differences were observed across race, payer, hospital location/teaching status, and hospital region, likely reflecting the large sample size rather than clinical significance (Table 2).

### 3.3. Postoperative Outcomes (Unadjusted Analysis)

Table 3 presents the unadjusted weighted outcomes. Steroid users had higher crude rates of any postoperative complication (6.8% vs. 4.2%; *p* < 0.001), major bleeding (3.9% vs. 2.2%; *p* < 0.001), acute kidney injury (2.8% vs. 1.9%; *p* = 0.034), and prolonged hospitalization (30.6% vs. 24.3%; *p* < 0.001). No significant differences were found for venous thromboembolism, sepsis, myocardial infarction, stroke, high hospital charges, discharge to rehabilitation, or mortality.

### 3.4. Univariate Odds Ratios

In unadjusted logistic regression (Table 4), chronic corticosteroid use was associated with significantly higher odds of any postoperative complication (OR 1.66, 95% CI 1.30–2.10), major bleeding (1.75, 1.28–2.39), acute kidney injury (1.52, 1.06–2.18), and prolonged hospitalization (1.37, 1.20–1.56). No significant associations were observed for venous thromboembolism, high hospital charges, discharge to rehabilitation, or mortality.

### 3.5. Multivariable Adjusted Analysis

After adjustment for demographic, clinical and hospital-level variables, chronic corticosteroid use remained significantly associated with several adverse outcomes (Table 4). Adjusted odds were highest for mortality (adjusted OR 2.53, 95% CI 1.02–6.31), major bleeding (1.46, 1.26–1.68), any complication (1.32, 1.18–1.47), and prolonged length of stay (1.26, 1.19–1.34). However, in-hospital mortality was a rare event (160 weighted deaths overall, including only 5 among chronic steroid users), and the wide confidence interval for the adjusted estimate reflects this small event count; this result should be regarded as imprecise and hypothesis-generating rather than a precise estimate of mortality risk. A smaller but statistically significant increase was also observed for discharge to rehabilitation (1.06, 1.00–1.12), whereas venous thromboembolism, acute kidney injury, and high hospital charges were not significant after adjustment.

A smaller but statistically significant increase was also observed for discharge to rehabilitation (1.06, 1.00–1.12), whereas venous thromboembolism, acute kidney injury, and high hospital charges were not significant after adjustment.

### 3.6. Propensity Score-Matched Analysis

Following 1:1 nearest-neighbor propensity matching, 2158 patients were included (1079 steroid users and 1079 matched controls). Covariate balance was excellent, with standardized mean differences (SMD) < 0.1 for all variables. In the matched cohort, chronic steroid use remained significantly associated with any complication (OR 1.84, 95% CI 1.24–2.72) and acute kidney injury (OR 2.10, 1.13–3.91). Associations with major bleeding, VTE, prolonged hospitalization, high hospital charges, and mortality were not statistically significant in the matched cohort. After false discovery rate correction, only any complication remained statistically significant (q = 0.020). The pattern of attenuation differed across outcomes. The association with any postoperative complication, the prespecified primary outcome, was directionally and statistically consistent across the unadjusted, adjusted, and matched analyses and remained significant after correction for multiple testing, supporting it as the most robust finding of this study. Acute kidney injury was significant in the matched cohort but not in the multivariable-adjusted model or after FDR correction, and should therefore be interpreted cautiously. Associations with major bleeding and prolonged length of stay, although significant in the unadjusted and adjusted analyses, were attenuated to no significance after matching, suggesting these effects may be partly explained by comorbidity imbalance that propensity matching addressed more completely than regression adjustment alone.

In a sensitivity analysis controlling the false discovery rate (Benjamini–Hochberg), only the association with any complication remained statistically significant after correction for multiple testing, whereas other outcomes did not retain significance. These findings support the robustness of the overall complication signal.

**Figure 1:**
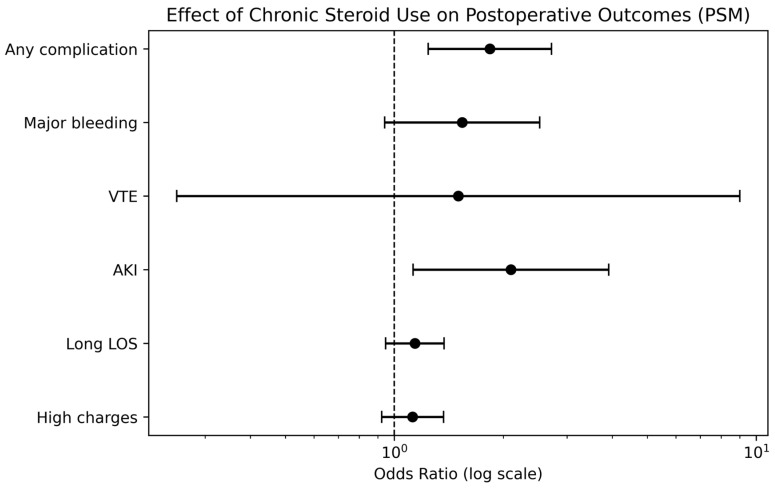
Effect of Chronic Steroid Use on Postoperative Outcomes (Propensity-Matched Cohort).

A forest plot illustrating the odds ratios (95% confidence intervals) for major postoperative outcomes among patients with chronic corticosteroid use compared with matched controls in the propensity score-matched cohort was constructed, corresponding to the estimates reported in Table 5; these are distinct from the weighted, multivariable-adjusted odds ratios presented in Table 4. Odds ratios are displayed on a logarithmic scale; the dashed line indicates the null value (OR = 1.0). Any complication and acute kidney injury were nominally significant in the matched cohort, while only any complication remained significant after false discovery rate correction. In-hospital mortality is not displayed because of its extremely wide confidence interval in the matched cohort (Figure 1).

### 3.7. Hospital Outcomes

Steroid users had slightly higher median hospital charges ($56,016 [IQR $40,290–$80,442] vs. $55,357 [IQR $39,703–$79,580]; *p* = 0.944) and longer mean length of stay (2.20 ± 1.61 vs. 2.00 ± 1.98 days; *p* < 0.001). (Table 6).

## 4. Discussion

In this large, nationally representative cohort of approximately 600,000 elective primary THA hospitalizations, chronic corticosteroid use—present in fewer than 1% of patients—was consistently associated with increased perioperative morbidity. Steroid users exhibited higher crude rates of postoperative complications, and after multivariable adjustment, chronic steroid therapy remained an independent predictor of overall complications, driven primarily by major bleeding and prolonged hospitalization. In propensity score-matched analyses, the association with any complication remained significant, whereas the signals for major bleeding and prolonged hospitalization were no longer statistically significant, indicating that these secondary findings are less robust than the primary complication outcome. While absolute differences in hospital charges were modest, steroid users demonstrated longer hospitalization. Collectively, these findings indicate that even though chronic corticosteroid exposure is uncommon among patients undergoing elective THA, it represents a clinically meaningful risk factor for heightened perioperative morbidity.

Chronic systemic corticosteroid therapy has been consistently associated with increased perioperative morbidity following elective total joint arthroplasty, and our findings align with this broader evidence base. Large cohort studies and meta-analyses have demonstrated higher rates of postoperative complications, readmissions, infectious events including periprosthetic joint infection, and reoperation among chronic steroid users compared with non-users [2,3,7,9]. In agreement with these reports, our nationally representative analysis of approximately 600,000 elective primary THA hospitalizations showed that patients on chronic corticosteroids experienced significantly higher crude rates of overall complications (6.8% vs. 4.2%), major bleeding (3.9% vs. 2.2%), acute kidney injury (2.8% vs. 1.9%), and prolonged hospitalization (30.6% vs. 24.3%). After multivariable adjustment, chronic steroid use remained an independent predictor of any postoperative complication (aOR 1.32), major bleeding (aOR 1.46), and prolonged hospitalization (aOR 1.26), with the association for any complication persisting in propensity score-matched analyses (OR 1.84). Prior studies have also shown that chronic corticosteroid therapy confers a dose-dependent risk of infection and readmission, particularly among patients with inflammatory conditions such as IBD [2,4]; although the NIS lacks dose granularity, our findings mirror this heightened vulnerability, as steroid users exhibited a greater comorbidity burden, including hypertension, COPD, obstructive sleep apnea, chronic anemia, and congestive heart failure, all factors that may potentiate adverse outcomes [9,10]. Importantly, whereas chronic corticosteroid exposure increases postoperative risk, short-term perioperative corticosteroid administration for analgesia or antiemesis in otherwise healthy arthroplasty patients does not appear to increase infectious or thromboembolic complications [5,8,11], highlighting that the exposure captured in the present study differs substantially from short-term perioperative steroid use. Collectively, these data reinforce that chronic corticosteroid use should be recognized as a marker of elevated perioperative risk in THA candidates, underscoring the need for further studies to determine whether targeted risk-stratification or management strategies can improve outcomes in this population.

Although the prior literature has generally suggested that chronic systemic corticosteroid therapy is not associated with increased perioperative major bleeding or transfusion requirements in elective total hip or knee arthroplasty [3,7,9,12], our findings indicate that steroid-dependent patients may, in fact, experience a higher bleeding risk than previously appreciated. Several retrospective studies and meta-analyses have reported no significant difference in intraoperative transfusion rates or major bleeding between chronic steroid users and matched controls, suggesting that any effect of long-term steroid exposure on hemostasis is minimal [13]. Similarly, short-term perioperative corticosteroid administration for pain or nausea control in otherwise healthy arthroplasty patients has not been linked to increased bleeding complications [5,8]. However, in our large national cohort of approximately 600,000 elective THA hospitalizations, chronic corticosteroid users had substantially higher crude rates of major bleeding (3.9% vs. 2.2%), and chronic steroid use remained an independent predictor of major bleeding after multivariable adjustment (aOR 1.46). This association was attenuated in propensity score-matched analyses. These results suggest that while bleeding risk in non-steroid users is primarily influenced by factors such as preoperative anemia, comorbidity burden, intraoperative blood loss, and drain use [14,15,16], chronic corticosteroid therapy may contribute an additional, clinically relevant risk factor in the arthroplasty population, particularly in medically complex patients. Further research is warranted to reconcile differences between prior studies and our findings, and to determine whether specific steroid doses, durations, or coexisting immunosuppressive therapies modify bleeding risk in elective THA.

Chronic systemic corticosteroid use has been repeatedly associated with an elevated risk of postoperative venous thromboembolism (VTE), including both deep vein thrombosis and pulmonary embolism, in patients undergoing elective total hip or knee arthroplasty. Large cohort studies and meta-analyses have demonstrated significantly increased odds of postoperative VTE among chronic steroid users, with adjusted odds ratios for DVT ranging from 2.07 to 2.39 and for PE up to 5.94, independent of baseline comorbidities [3,7,17]. In the present analysis, VTE was uncommon and did not differ significantly between steroid users and non-users (0.3% vs. 0.2%). Chronic steroid exposure was not an independent predictor of postoperative VTE after adjustment (aOR 1.20), and the association was not significant in the propensity score-matched cohort. Prior evidence suggests that thromboembolic risk may be most pronounced in patients with recent initiation of systemic steroids and higher cumulative doses [17], which could not be captured in our administrative dataset. More broadly, because chronic steroid use in this cohort is itself a marker of underlying inflammatory or autoimmune disease, frailty, and other immunosuppressive therapies, any residual association between steroid exposure and thromboembolic risk could reflect confounding by these unmeasured factors rather than a direct pharmacologic effect. VTE was not significantly associated with steroid use in any of the unadjusted, adjusted, or matched analyses in our cohort, which may itself partly reflect the rarity of the outcome and limited statistical power, underscoring the need for studies with more granular clinical and pharmacologic data. In contrast, short-term perioperative corticosteroid administration such as dexamethasone used for analgesia, nausea control, or enhanced recovery protocols does not increase, and may even reduce, the risk of postoperative VTE, as demonstrated in randomized trials and large observational studies [8,18,19,20,21]. This distinction reinforces that VTE risk may depend on chronic exposure patterns, underlying disease burden, and patient selection rather than single-dose perioperative corticosteroid use.

Chronic systemic corticosteroid therapy also appears to influence hospital resource utilization following arthroplasty. Consistent with earlier studies linking chronic steroid use to higher comorbidity burden, increased ASA classification, and greater postoperative complication risk [3,7,17,18], we found that steroid users undergoing elective THA experienced longer hospital stays (2.20 vs. 2.00 days) and were more likely to have prolonged lengths of stay (aOR 1.26). This association was attenuated and was not statistically significant in propensity score-matched analyses. Chronic steroid users were also more frequently discharged to rehabilitation facilities after adjustment (aOR 1.06), likely reflecting the additive influence of complications, delayed recovery, and baseline medical complexity [3,19]. Importantly, although perioperative corticosteroids in otherwise healthy patients have been associated with enhanced recovery and reduced length of stay [8,20,21], these benefits do not extend to individuals on chronic corticosteroid therapy, for whom preexisting immunosuppression and comorbidities drive increased perioperative risk and resource needs. Collectively, these findings underscore the importance of distinguishing between chronic steroid exposure and short-term perioperative administration when evaluating patient risk profiles and postoperative care pathways.

Preoperative chronic systemic corticosteroid use is consistently associated with increased postoperative resource utilization in adults undergoing elective primary total hip arthroplasty, and our findings support this association. Prior studies have shown that chronic steroid users experience longer hospitalization and are more frequently discharged to rehabilitation facilities, largely due to higher rates of postoperative complications and a greater burden of medical comorbidities [3,7,17,18]. Elevated ASA classification and increased multimorbidity, both of which are more prevalent among chronic steroid users, have been strongly linked to prolonged length of stay after THA [3,19]. In alignment with this literature, our national cohort demonstrated that chronic corticosteroid users had a longer mean hospital stay (2.20 vs. 2.00 days) and significantly higher odds of prolonged length of stay after adjustment (aOR 1.26). Steroid users were also more likely to be discharged to rehabilitation facilities after adjustment (aOR 1.06), emphasizing the increased postoperative care needs in this population.

It is important to distinguish the effects of chronic preoperative corticosteroid exposure from those of short-term perioperative corticosteroid administration. While perioperative steroids administered for analgesia, nausea control, or enhanced recovery protocols are associated with reduced length of stay and improved postoperative recovery in otherwise healthy arthroplasty patients [8,20,21], these benefits do not extend to individuals on chronic corticosteroid therapy. Instead, chronic steroid use serves as a marker of heightened perioperative risk, greater comorbidity burden, and increased likelihood of requiring extended postoperative care and rehabilitation resources.

Chronic systemic corticosteroid use is well recognized as a risk factor for adverse postoperative outcomes, with multiple studies demonstrating higher rates of infection, impaired wound healing, adrenal insufficiency, and increased overall perioperative morbidity in steroid-dependent patients undergoing major surgery, including total hip arthroplasty [22,23]. Our findings align with this established risk profile: chronic corticosteroid users in our national THA cohort experienced significantly greater odds of overall complications (aOR 1.32), major bleeding (aOR 1.46), and prolonged hospitalization (aOR 1.26), as well as a greater likelihood of discharge to rehabilitation facilities. In response to these well-documented risks, current guidelines from the American College of Rheumatology/American Association of Hip and Knee Surgeons and the European Society of Endocrinology recommend continuation of a patient’s baseline corticosteroid regimen throughout the perioperative period, reserving stress-dose supplementation for individuals exhibiting hemodynamic instability or clinical indicators of adrenal crisis, and adjusting postoperative dosing according to expected surgical stress [22,23,24]. Key strategies for risk mitigation include careful monitoring for perioperative adrenal insufficiency, judicious administration of stress-dose corticosteroids in patients at risk of hypothalamic–pituitary–adrenal axis suppression, and optimization of adherence to guideline-directed perioperative management [23,25]. While perioperative corticosteroid administration for analgesia or nausea control in otherwise healthy arthroplasty patients has not been shown to increase postoperative infection or thromboembolic risk [5,6,26], chronic corticosteroid users remain uniquely susceptible to hyperglycemia, infectious complications, impaired tissue healing, and prolonged hospitalization, with risks increasing in a dose-dependent manner [22]. Despite broad consensus on core principles of perioperative management, significant evidence gaps persist regarding optimal steroid supplementation protocols, dose–response relationships, and long-term outcomes in this high-risk population [23,24,25]. Taken together, our findings identify chronic corticosteroid use as a marker of elevated perioperative risk, lending support to the rationale underlying existing guideline recommendations for baseline therapy continuation and individualized stress-dose supplementation. However, because this retrospective database analysis could not evaluate the effectiveness of any specific management or monitoring strategy, prospective studies are needed to determine whether such approaches measurably reduce perioperative risk in this population.

Although total hip arthroplasty remains the definitive treatment for end-stage hip degeneration, conservative and joint-preserving strategies may be considered in selected patients, particularly those with early-stage osteonecrosis or less advanced disease. Recent studies have highlighted the potential role of nonoperative management and hip-preserving procedures in delaying disease progression and postponing the need for arthroplasty in carefully selected individuals. Nevertheless, patients who progress to femoral head collapse or advanced joint destruction frequently require THA to restore function and alleviate pain. Therefore, conservative treatment should be viewed as part of a continuum of care, with arthroplasty reserved for patients in whom joint-preserving measures fail or are no longer appropriate [27,28].

This study has several limitations inherent to its retrospective design and reliance on administrative claims data. First, the National Inpatient Sample lacks clinical detail regarding corticosteroid dose, duration, indication, and degree of hypothalamic–pituitary–adrenal axis suppression, preventing dose–response assessments and limiting granularity in characterizing steroid exposure. Second, complications were identified using ICD-10 codes during the index hospitalization only; therefore, events occurring after discharge such as 30- or 90-day venous thromboembolism, infection, or readmission were not captured, likely leading to underestimation of true complication rates. Third, although extensive multivariable adjustment and propensity score matching were used to reduce confounding, residual confounding from unmeasured factors such as frailty, nutritional status, smoking, steroid taper patterns, immunosuppressive co-medications, and perioperative management practices cannot be excluded. Fourth, some variables, including obesity, had high levels of missing data and were excluded from adjusted models, which may have influenced effect estimates. Additionally, because perioperative steroid supplementation and stress-dose regimens are not recorded in the NIS, we could not determine whether adverse outcomes were related to inadequate or excessive perioperative steroid replacement. Additionally, because in-hospital mortality and several other complications (myocardial infarction, stroke, sepsis) were rare events in this cohort, the study was underpowered to estimate these associations precisely. The mortality estimate in particular, despite reaching nominal statistical significance in the adjusted model, should be interpreted cautiously given the small number of events and the wide confidence interval, rather than as a precise or definitive estimate of perioperative mortality risk. For these and other low-event secondary outcomes, nonsignificant findings should be interpreted as an absence of evidence for an association rather than evidence that no true association exists, given the limited statistical power to detect clinically meaningful effects. Finally, as with all large database studies, miscoding and misclassification remain possible despite the use of validated coding algorithms; in particular, major bleeding, sepsis, and venous thromboembolism were identified using ICD-10 diagnostic and procedural codes recorded only during the index hospitalization, so both under-ascertainment (e.g., events managed nonoperatively, undercoded, or diagnosed after discharge) and coding variability across hospitals are possible, which would tend to bias estimates toward the null and could lead to underestimation of true event rates for these outcomes. These limitations should be considered when interpreting the findings, although the large, nationally representative cohort and consistency across multiple analytic approaches strengthen the validity of the observed associations.

## 5. Conclusions

In conclusion, chronic systemic corticosteroid use, although uncommon among patients undergoing elective primary total hip arthroplasty, is associated with a clinically meaningful increase in perioperative morbidity. Steroid-dependent patients demonstrated higher risks of overall complications across all analytic approaches, including after propensity score matching and correction for multiple testing. Associations with major bleeding, acute kidney injury, and prolonged hospitalization were also observed in unadjusted and/or adjusted analyses, but were less consistent after matching, and should be regarded as hypothesis-generating rather than definitively established. While the absolute differences in outcomes were modest, the consistency of these findings across analytic approaches underscores the need for greater clinical recognition of chronic corticosteroid use as a perioperative risk factor in this population. Because this retrospective database study did not evaluate any specific management strategy, these findings should be regarded as hypothesis-generating; further research is warranted to clarify dose–response relationships, evaluate perioperative steroid supplementation protocols, and determine whether targeted risk-mitigation strategies can improve outcomes for patients requiring chronic corticosteroid therapy.

## Figures and Tables

**Table 1:** ICD-10 Codes Used for Exposure, Comorbidities, and Postoperative Complications.

Category	Variable	ICD-10-CM/PCS Codes	Definition/Notes
Exposure	Chronic corticosteroid use	Z79.52	Long-term (current) use of systemic corticosteroids
Comorbidities	Hypertension	I10–I15	Primary and secondary hypertension
	Obesity	E66 *	All obesity codes
	Diabetes mellitus	E10–E14	Includes type 1 and type 2 diabetes, with or without complications
	Dyslipidemia	E78 *	Disorders of lipoprotein metabolism and other lipidemias
	Chronic kidney disease	N18 *	Chronic renal failure and stages 1–5 CKD
	Obstructive sleep apnea (OSA)	G47.33	Obstructive sleep apnea
	Chronic anemia	D64 *	Other anemias (non-nutritional)
	Alcohol abuse	F10 *	Mental and behavioral disorders due to alcohol use
	Chronic lung disease/COPD	J40–J44, J47	Includes chronic bronchitis, emphysema, and bronchiectasis
	Congestive heart failure (CHF)	I50 *	Heart failure, all types
Postoperative complications	Major bleeding (diagnosis)	T81.0 *	Hemorrhage and hematoma complicating a procedure
	Major bleeding (transfusion)	30233N1, 30233P1, 30233R1, 30243N1, 30243P1, 30243R1	ICD-10-PCS codes indicating blood transfusion
	Deep vein thrombosis (DVT)	I82.4 *, I82.5 *, I82.9 *	Acute venous embolism and thrombosis of lower extremities or unspecified sites
	Pulmonary embolism (PE)	I26 *	Acute pulmonary embolism
	Venous thromboembolism (VTE)	—	Defined as DVT or PE
	Wound dehiscence	T81.3 *	Disruption of operation wound
	Sepsis	A41 *, T81.44 *	Sepsis and postoperative septic shock
	Acute kidney injury (AKI)	N17 *	Acute renal failure and subcategories
	Myocardial infarction (MI)	I21 *	Acute myocardial infarction
	Stroke/CVA	I63 *, I64 *	Cerebral infarction and unspecified stroke
	In-hospital mortality	—	Defined by discharge disposition (DIED = 1)

* Includes all codes starting with the indicated prefix (e.g., E66 * includes all E66 subcodes).

**Table 2:** Baseline patient and hospital characteristics by chronic corticosteroid use.

Variable	Overall (N = 599,720)	No Steroids (N = 594,265)	Steroids (N = 5455)	*p*-Value
**Age (mean ± SD)**	65.1 ± 11.1	65.1 ± 11.1	63.8 ± 13.8	*0.0013*
**Female, n (%)**	328,850 (54.83%)	325,535 (54.78%)	3315 (60.77%)	*<0.001*
**Race, n (%)**				*0.0011*
White	488,700 (83.95%)	484,585 (84.01%)	4115 (77.35%)	
Black	53,835 (9.25%)	53,085 (9.20%)	750 (14.10%)	
Hispanic	22,310 (3.83%)	22,045 (3.82%)	265 (4.98%)	
Asian or Pacific Islander	4675 (0.80%)	4620 (0.80%)	55 (1.03%)	
Native American	1600 (0.27%)	1555 (0.27%)	45 (0.85%)	
Other	11,005 (1.89%)	10,915 (1.89%)	90 (1.69%)	
**Primary Payer, n (%)**				*<0.001*
Medicare	317,020 (52.95%)	313,795 (52.90%)	3225 (59.17%)	
Medicaid	33,075 (5.52%)	32,740 (5.52%)	335 (6.15%)	
Private insurance	227,375 (37.98%)	225,650 (38.04%)	1725 (31.65%)	
Self-pay	4730 (0.79%)	4680 (0.79%)	50 (0.92%)	
No charge	380 (0.06%)	375 (0.06%)	5 (0.09%)	
Other	16,085 (2.69%)	15,975 (2.69%)	110 (2.02%)	
**Comorbidities, n (%)**				
Hypertension	58,075 (9.68%)	57,385 (9.66%)	690 (12.65%)	*0.0011*
Obesity	24,050 (25.77%)	23,745 (25.73%)	305 (29.76%)	0.218
Diabetes	16,335 (2.72%)	16,150 (2.72%)	185 (3.39%)	0.205
Dyslipidemia	252,185 (42.05%)	249,850 (42.04%)	2335 (42.80%)	*0.634*
Obstructive Sleep Apnea	60,075 (10.02%)	59,340 (9.99%)	735 (13.47%)	*<0.001*
Chronic Anemia	32,490 (5.42%)	32,030 (5.39%)	460 (8.43%)	*<0.001*
Alcohol Abuse	8780 (1.46%)	8715 (1.47%)	65 (1.19%)	*0.531*
COPD	44,300 (7.39%)	43,530 (7.33%)	770 (14.12%)	*<0.001*
CHF	17,340 (2.89%)	17,015 (2.86%)	325 (5.96%)	*<0.001*
**Hospital Characteristics**				
Hospital Bed Size, n (%)				*0.144*
Small	174,465 (29.09%)	172,995 (29.11%)	1470 (26.95%)	
Medium	174,610 (29.12%)	173,055 (29.12%)	1555 (28.51%)	
Large	250,645 (41.79%)	248,215 (41.77%)	2430 (44.55%)	
**Hospital Location/Teaching, n (%)**				*0.0025*
Rural	36,815 (6.14%)	36,490 (6.14%)	325 (5.96%)	
Urban nonteaching	135,555 (22.60%)	134,555 (22.64%)	1000 (18.33%)	
Urban teaching	427,350 (71.26%)	423,220 (71.22%)	4130 (75.71%)	
**Hospital Region, n (%)**				*0.0076*
Northeast	122,620 (20.45%)	121,695 (20.48%)	925 (16.96%)	
Midwest	127,265 (21.22%)	126,140 (21.23%)	1125 (20.62%)	
South	236,395 (39.42%)	234,010 (39.38%)	2385 (43.72%)	
West	113,440 (18.92%)	112,420 (18.92%)	1020 (18.70%)	

Values are weighted proportions. Differences tested using *t*-tests for continuous variables and χ^2^ tests for categorical variables.

**Table 3:** Unadjusted postoperative outcomes by chronic corticosteroid use.

Outcome	Overall (N = 599,720)	No Steroids (N = 594,265)	Steroids (N = 5455)	*p*-Value
Any complication	25,305 (4.22%)	24,935 (4.20%)	370 (6.78%)	<0.001
Major bleeding	13,310 (2.22%)	13,100 (2.20%)	210 (3.85%)	<0.001
Venous thromboembolism	1245 (0.21%)	1230 (0.21%)	15 (0.27%)	0.498
Acute kidney injury	11,500 (1.92%)	11,345 (1.91%)	155 (2.84%)	0.034
Sepsis	440 (0.07%)	435 (0.07%)	5 (0.09%)	0.553
Myocardial infarction	545 (0.09%)	545 (0.09%)	0 (0.00%)	0.630
Stroke	360 (0.06%)	360 (0.06%)	0 (0.00%)	>0.99
Prolonged LOS (>75th percentile)	145,765 (24.31%)	144,095 (24.25%)	1670 (30.61%)	<0.001
High hospital charges (≥75th percentile)	149,085 (24.86%)	147,695 (24.85%)	1390 (25.48%)	0.658
Discharge to rehabilitation (%)	350,930 (58.52%)	347,645 (58.50%)	3285 (60.22%)	0.264
In-hospital mortality (%)	160 (0.03%)	155 (0.03%)	5 (0.09%)	0.254

**Table 4:** Unadjusted and Adjusted odds ratios for postoperative outcomes.

Outcome	OR (95% CI)	*p*-Value	Adjusted OR (95% CI)	*p*-Value
Any complication	1.66 (1.30–2.10)	<0.001	1.32 (1.18–1.47)	<0.001
Major bleeding	1.75 (1.28–2.39)	<0.001	1.46 (1.26–1.68)	<0.001
VTE	1.33 (0.43–4.17)	0.497	1.20 (0.72–1.99)	0.495
AKI	1.52 (1.06–2.18)	0.028	1.12 (0.95–1.33)	0.184
Long LOS	1.37 (1.20–1.56)	<0.001	1.26 (1.19–1.34)	<0.001
High charges	1.03 (0.89–1.18)	0.746	1.00 (0.93–1.07)	0.986
Discharge to rehab	1.07 (0.95–1.21)	0.286	1.06 (1.00–1.12)	0.036
Mortality	3.61 (0.49–26.52)	0.248	2.53 (1.02–6.31)	0.046

**Table 5:** Propensity Score-Matched Analysis of Postoperative Outcomes.

Outcome	PSM OR (95% CI)	*p*-Value
**Any complication**	1.84 (1.24–2.72)	0.0029
**Major bleeding**	1.54 (0.94–2.52)	0.109
**VTE**	1.50 (0.25–9.00)	>0.99
**AKI**	2.10 (1.13–3.91)	0.025
**Long LOS**	1.14 (0.95–1.37)	0.184
**High charges**	1.12 (0.92–1.37)	0.269
**Mortality**	3.00 (0.12–73.80)	>0.99

**Table 6:** Comparison of hospitalization outcomes between patients with and without chronic corticosteroid use undergoing total hip arthroplasty.

Outcome	Overall (N = 599,720)	No Steroids (N = 594,265)	Steroids (N = 5455)	*p*-Value
**Total Charges ($)**	55,366.00 [39,712–79,587]	55,357.00 [39,703–79,580]	56,016.00 [40,290–80,442]	0.944
**Length of Stay (days)**	2.00 ± 1.97	2.00 ± 1.98	2.20 ± 1.61	<0.001

## Data Availability

The data that support the findings of this study are available from the Healthcare Cost and Utilization Project (HCUP) National Inpatient Sample (NIS) database. Restrictions apply to the availability of these data, which were used under license for the current study and are therefore not publicly available. Data are available from HCUP upon reasonable request and completion of the required data use agreement.

## Abbreviations

The following abbreviations are used in this manuscript:

AHRQ	Agency for Healthcare Research and Quality
AKI	Acute Kidney Injury
aOR	Adjusted Odds Ratio
ASA	American Society of Anesthesiologists
CHF	Congestive Heart Failure
CI	Confidence Interval
CKD	Chronic Kidney Disease
CM	Clinical Modification
COPD	Chronic Obstructive Pulmonary Disease
CVA	Cerebrovascular Accident
DISCWT	Discharge Weight
DVT	Deep Vein Thrombosis
HCUP	Healthcare Cost and Utilization Project
IBD	Inflammatory Bowel Disease
ICD-10-CM	International Classification of Diseases, Tenth Revision, Clinical Modification
ICD-10-PCS	International Classification of Diseases, Tenth Revision, Procedure Coding System
IQR	Interquartile Range
LOS	Length of Stay
MI	Myocardial Infarction
NIS	National Inpatient Sample
OR	Odds Ratio
OSA	Obstructive Sleep Apnea
PCS	Procedure Coding System
PE	Pulmonary Embolism
PJI	Prosthetic Joint Infection
PSM	Propensity Score Matching
SD	Standard Deviation
SMD	Standardized Mean Difference
SSI	Surgical Site Infection
THA	Total Hip Arthroplasty
VTE	Venous Thromboembolism
